# Enhanced Oxygen Reduction Reaction by In Situ Anchoring Fe_2_N Nanoparticles on Nitrogen-Doped Pomelo Peel-Derived Carbon

**DOI:** 10.3390/nano7110404

**Published:** 2017-11-22

**Authors:** Yiqing Wang, Mingyuan Zhu, Gang Wang, Bin Dai, Feng Yu, Zhiqun Tian, Xuhong Guo

**Affiliations:** 1 Key Laboratory for Green Processing of Chemical Engineering of Xinjiang Bingtuan, School of Chemistry and Chemical Engineering, Shihezi University, Shihezi 832003, China; wangyiqing_yes@sina.com (Y.W.); zhuminyuan@shzu.edu.cn (M.Z.); wanggang@shzu.edu.cn (G.W.); db_tea@shzu.edu.cn (B.D.); 2 Engineering Research Center of Materials-Oriented Chemical Engineering of Xinjiang Production and Construction Corps, Shihezi 832003, China; 3 Key Laboratory of Materials-Oriented Chemical Engineering of Xinjiang Uygur Autonomous Region, Shihezi 832003, China; 4 Collaborative Innovation Center of Renewable Energy Materials, Guangxi University, Nanning 530004, China; 5 State Key Laboratory of Chemical Engineering, East China University of Science and Technology, Shanghai 200237, China

**Keywords:** Fe_2_N electrocatalyst, pomelo peel, oxygen reduction reaction, biomass, agricultural waste

## Abstract

The development of effective oxygen electrode catalysts for renewable energy technologies such as metal-air batteries and fuel cells remains challenging. Here, we prepared a novel high-performance oxygen reduction reaction (ORR) catalyst comprised of Fe_2_N nanoparticles (NPs) in situ decorated over an N-doped porous carbon derived from pomelo peel (i.e., Fe_2_N/N-PPC). The decorated Fe_2_N NPs provided large quantities of Fe-N-C bonding catalytic sites. The as-obtained Fe_2_N/N-PPC showed superior onset and half-wave potentials (0.966 and 0.891 V, respectively) in alkaline media (0.1 M KOH) compared to commercial Pt/C through a direct four-electron reaction pathway. Fe_2_N/N-PPC also showed better stability and methanol tolerance than commercial Pt/C. The outstanding ORR performance of Fe_2_N/N-PPC was attributed to its high specific surface area and the synergistic effects of Fe_2_N NPs. The utilization of agricultural wastes as a precursor makes Fe_2_N/N-PPC an ideal non-precious metal catalyst for ORR applications.

## 1. Introduction

According to the United Nations, almost 140 billion metric tons of biomass is generated globally from agricultural wastes. Therefore, the valorization of biomass can potentially reduce the use of fossil fuels while mitigating greenhouse gas emissions [1]. Biomass wastes, such as bananas [2,3], tea leaves [4], poplar catkins [5], gingko leaves [6], coconut shells [1], eggshell membranes [7], and sodium alginate [8], have been increasingly used for fabricating carbon materials. Biomass raw materials can be used to synthesize carbon materials with a high surface area and porous structure. This approach possesses several advantages (e.g., low cost, environmental friendliness, and convenience) over other routes employing different raw materials. Thus, biomass represents a nearly limitless resource for preparing a variety of new materials with novel structures and properties. Biomass-derived carbons have been employed as environmental adsorption materials [9] and lithium-ion battery anodes [10], among other uses [11].

Pomelo is widely cultivated and generates large amounts pomelo peels as an environmental waste. It represents a sustainable, inexpensive, and abundant resource supplied by nature [12]. Pomelo peels have been used to prepare porous carbons for supercapacitor [13,14] or adsorption applications [15]. Pomelo peels have been successfully used as a raw material to prepare functional carbon materials with oxygen reduction reaction (ORR) properties. Thus, Yuan et al. synthesized waste pomelo peel-derived N-doped nanoporous carbons as metal-free ORR electrocatalysts [4]. Wang et al. synthesized ZIF-67-containing carbon catalysts derived from pomelo peels with high efficiency towards the ORR [16]. Ma et al. synthesized pomelo peel-derived porous Fe_3_C/tungsten carbide (WC)/graphitic carbon nanocomposites with high electrocatalytic activity [17]. Compared with other biomass residues, pomelo peel-derived carbon (PPC) materials show better developed porosities and higher specific surface area [16].

A large variety of ORR electrocatalysts are currently being investigated since this process is a key limiting factor for developing highly efficient fuel cells [18,19]. Pt is usually used as the ORR catalyst, although it suffers from several issues (e.g., high price, poor stability, susceptibility to methanol crossover, and carbon monoxide poisoning), which hinders practical application and commercialization of fuel cells [20]. The identification of cheap and environmentally friendly precursors and green synthesis routes leading to ORR catalysts at an industrial scale remains challenging. Therefore, economically viable, efficient, and durable alternative electrocatalysts, such as noble metal-free [21,22,23,24,25] or metal-free [26,27] materials, are highly desirable for fuel cells to penetrate the market owing to their low cost, easy preparation, and high catalytic activity [22,28]. Ternary Fe/N/C materials are one of the most promising candidates by virtue of their high ORR performance (resulting from the synergistic effect between N and Fe) and abundancy in the earth [4,29,30].

Herein, we developed a green and easy approach for preparing effective ORR catalysts with pomelo peel, ammonia and FeCl_3_·6H_2_O. We synthesized highly graphitic porous carbon from pomelo peel by carbonization. Subsequently, Fe-based nanoparticles (NPs) composed of Fe_2_N and other nanocrystalline phases were loaded onto N-doped pomelo peel-derived carbon (N-PPC) by calcining the PPC previously impregnated with a FeCl_3_·6H_2_O solution under NH_3_ atmosphere. The resultant Fe-N-PPC catalyst showed more positive onset and half-wave potentials (by 14 and 26 mV, respectively) versus a commercial Pt/C catalyst. The Fe-N-PPC catalyst also outperformed Pt/C in terms of stability and methanol tolerance under alkaline conditions.

## 2. Results and Discussion

**Figure 1:**
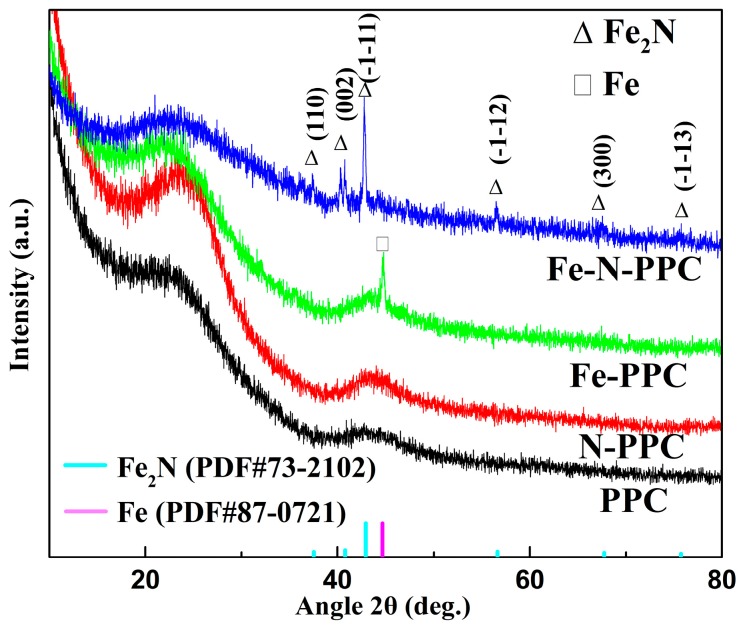
X-ray diffraction (XRD) patterns of PPC, N-PPC, Fe-PPC, and Fe-N-PPC carbonized at 900 °C.

With the aim of identifying the nanocrystalline phases present in the PPC, N-PPC, Fe-PPC, and Fe-N-PPC materials, X-ray diffraction (XRD) measurements were carried out (Figure 1). PPC and N-PPC only showed the two typical (002) and (100) peaks of graphitic carbon at 26.2 and 43.3°. Fe-PPC showed a minor amount of Fe (PDF#87-0721). The XRD patterns of Fe-PPC and Fe-N-PPC showed a broad peak at 23° a small shoulder peak at 43.3° assigned to the (002) (100) planes of graphite, respectively. A Fe_2_N crystalline phase (PDF#73-2102) was also detected in Fe-N-PPC. Moreover, the Fe-based NPs were accompanied by strong C signals, thereby demonstrating that these NPs were successfully loaded on the PPC material. The (002) diffraction peak shifted to lower angles (23°) for Fe-PPC and Fe-N-PPC as compared to PPC and N-PPC (26.2°), revealing an larger interplanar distance [31].

**Figure 2:**
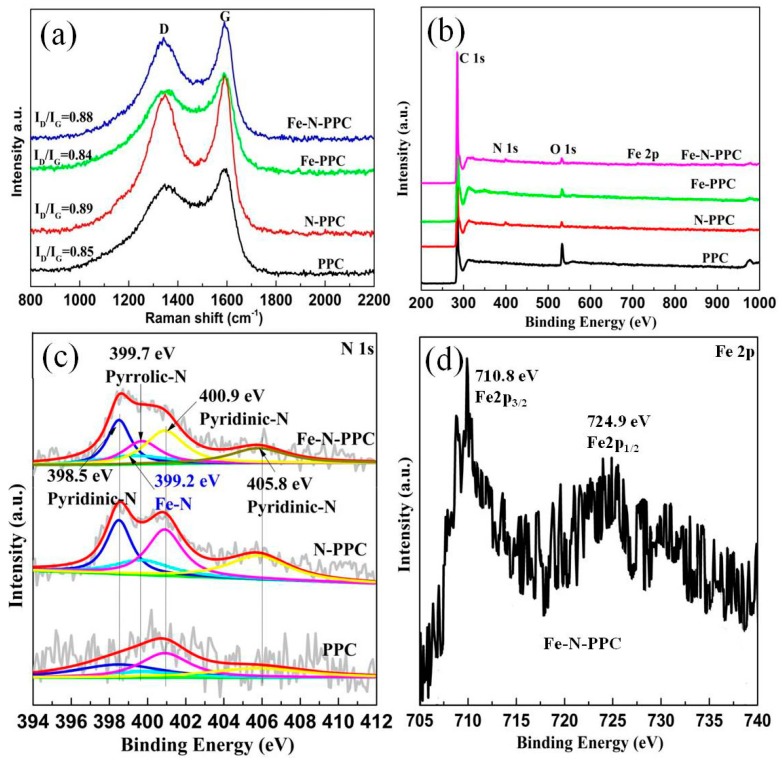
(**a**) Raman spectra and (**b**) High-resolution X-ray photoelectron spectroscopy (XPS) spectra of PPC, N-PPC, Fe-PPC, and Fe-N-PPC; (**c**) N 1s spectra of PPC, N-PPC and Fe-N-PPC; (**d**) Fe 2p spectra of Fe-N-PPC.

The I_D_/I_G_ intensity ratio values obtained from the Raman spectra were 0.85, 0.89, 0.84, and 0.88 for the PPC, N-PPC, Fe-PPC, and Fe-N-PPC samples, respectively (Figure 2a). The D and G Raman bands are indicative of sp^3^ defect sites of the graphite layers and the E_2g_ vibrational mode of sp^2^-bonded pairs, respectively. Thus, the I_D_/I_G_ intensity ratio values obtained revealed that the introduction of Fe and N generated defects and disorderliness in the porous carbon material. These defects and disorder favored the formation of active sites on the surface of the catalysts. The intensity of the G-band increased upon addition of Fe^3+^. The I_D_/I_G_ intensity ratio values remained nearly unchanged for all the samples, which resulted in excellent ORR activity.

XPS measurements were performed to analyze in detail elemental composition and chemical valence of PPC, N-PPC, N-PPC, and Fe-N-PPC. As shown in Figure 2b and Table 1, the XPS spectrum of Fe-N-PPC showed the presence of C (95.86%), N (1.60%), O (2.12%), and Fe (0.43%). The N 1s spectra was deconvoluted into five bands centered at 398.5, 399.2, 399.7, 400.9, and 405.8 eV (Figure 2c) assigned to pyridinic-N, N-Fe compounds, pyrrolic-N, graphitic-N, and oxidized-N, respectively [32]. PPC, N-PPC, and Fe-N-PPC showed pyridinic-N contents of 25.9 at. %, 31.9 at. %, and 32.2 at. %, respectively. Other N species contents are also listed in Table 2. Remarkably, the ORR active sites in the N-doped carbon nanomaterials were identified to involve carbon atoms with Lewis basicity and located in the vicinity of pyridinic-N [19]. Thus, the high content of pyridinic-N of the N-PPC and Fe-N-PPC sample is important in enhancing the ORR performance of these materials. The N-Fe compounds (9.0 at. %) showed the presence of iron nitride species. The Fe 2p spectra of Fe-N-PPC (Figure 2d) contained weak Fe 2p^3/2^ and Fe 2p^1/2^ doublet signals at 710.8 eV and at 724.9 eV, respectively, suggesting the presence of slightly metallic species [29,31,33,34,35,36].

**Figure 3:**
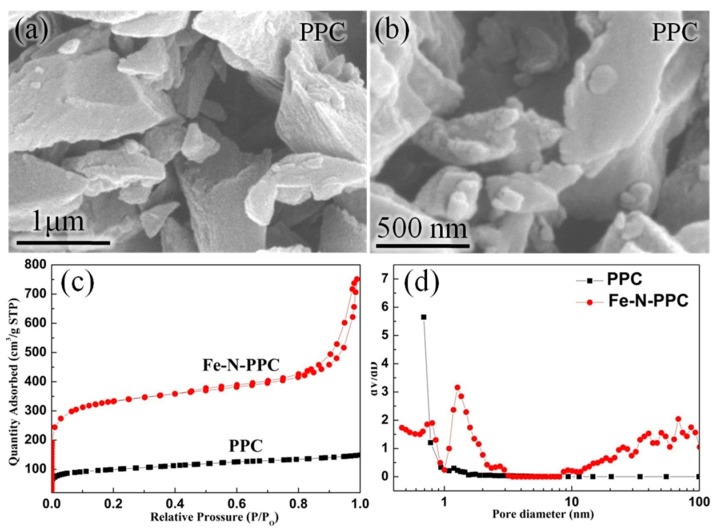
(**a**,**b**) Scanning electron microscopy (SEM) images of PPC; (**c**) N_2_ adsorption–desorption isotherms and (**d**) Pore size distributions of PPC and Fe-N-PPC respectively.

**Figure 4:**
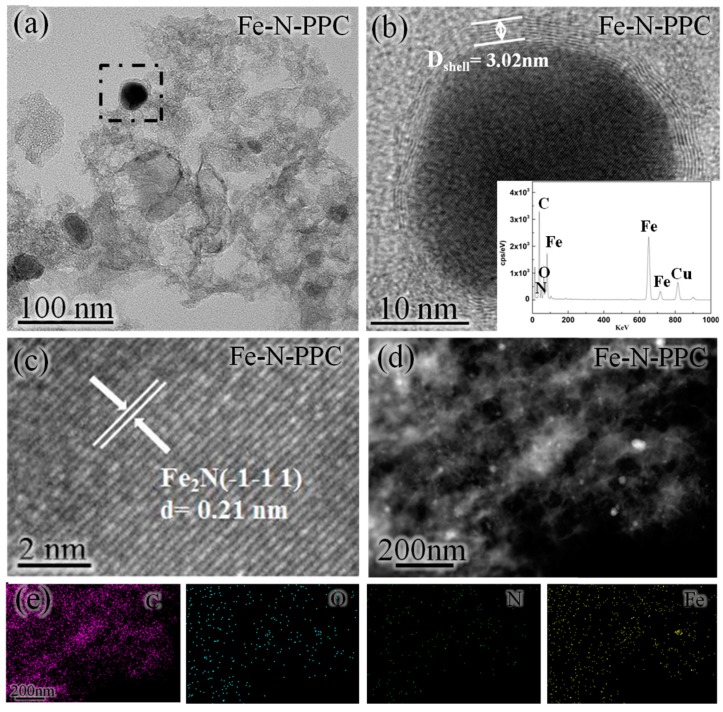
(**a**) Transmission electron microscopy (TEM) images; (**b**,**c**) energy dispersive X-ray spectroscopy (EDS) patterns and high resolution TEM (HRTEM) images of Fe-N-PPC; (**d**) high-angle annular dark-field scanning transmission electron microscopy (HAADF-STEM) image and (**e**) EDS elemental mapping images (C, O, N and Fe) of Fe-N-PPC.

The scanning electron microscopy (SEM) images of PPC are shown in Figure 3a,b. As indicated above, Fe-N-PPC was prepared from pomelo peel via a two-step carbonization process followed by calcination at 900 °C under an NH_3_ atmosphere. This calcination process resulted in Fe^3+^/PPC being converted into a Fe-N-PPC. As shown in Figure 4a,d, transmission electron microscopy (TEM) and high-angle annular dark-field scanning transmission electron microscopy (HAADF-STEM) observations revealed the presence of Fe_2_N NPs distributed inside or anchored on the N-doped porous carbon with little agglomeration. The size of the Fe_2_N NPs was in the 20–30 nm range. Figure 3c shows the N_2_ physisorption isotherms of the PPC and Fe-N-PPC catalysts. Fe-N-PPC exhibited a typical type IV curve characteristic of mesoporous materials. The pore size distribution curves (Figure 3d) of PPC and Fe-N-PPC revealed average pore sizes 2.60 and 3.48 nm, respectively. As shown in Table 3, Fe-N-PPC exhibited higher Brunauer-Emmett-Teller (BET) surface area than PPC (1103.90 versus 353.05 m^2^·g^−1^). We concluded that increasing the temperature of the calcination treatment to 900 °C was effective in increasing the surface area of the materials by generating numerous defects. Larger BET surface area and accompanying defects are important in improving the ORR catalytic activity [5,34]. Thus, Fe-N-PPC showed superior ORR performance.

As revealed by energy-dispersive energy dispersive X-ray spectroscopy (EDS), the content of elemental Fe in the Fe-N-PPC varied significantly after the double carbonization treatment (Figure 4b,e) [29]. EDS mapping analysis of Fe-N-PPC indicate the presence of C, O, N, and Fe atoms. As shown by high resolution TEM (HRTEM) (Figure 4b), graphitized carbon protected these NPs from agglomerating and the thickness of the carbon layer is about 3 nm [29,37], thereby improving the ORR activity. The HRTEM image of Fe_2_N NPs revealed a fringe spacing of 0.21 nm, which corresponded to the (−1−11) plane of the Fe_2_N crystal phase (Figure 4c).

**Figure 5:**
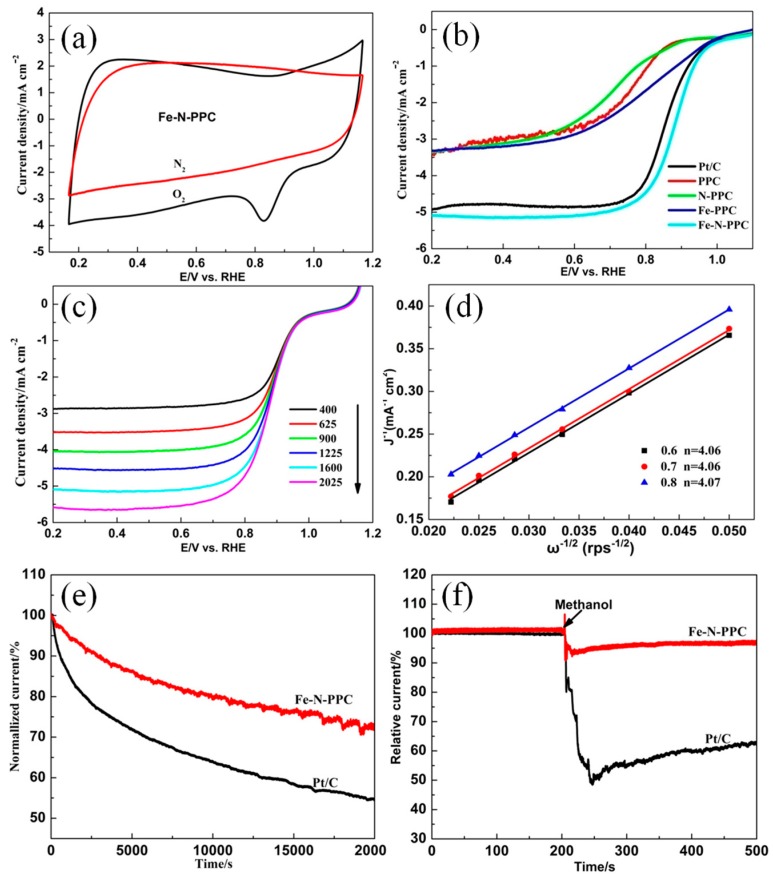
(**a**) The cyclic voltammetry (CV) curves of Fe-N-PPC at a scan of 50 mV/s; (**b**) LSV curves of PPC and other samples at a scan rate of 10 mV/s and an electrode rotation rate of 1600 rpm; (**c**) LSV curves of Fe-N-PPC at various rotation rates and a scan rate of 10 mV/s; (**d**) Corresponding K–L plots at different potentials; (**e**) Long-term stability test of Fe-N-PPC and 20% Pt/C in a 0.1 M KOH solution; (**f**) Methanol (3 M) crossover resistance test.

As shown in Figure 5a, Fe-N-PPC exhibited a noticeable reduction peak in the O_2_-saturated 0.1 M KOH solution, and this peak was absent in the N_2_-saturated 0.1 M KOH solution. These results are indicative of the high ORR electrocatalytic activity of Fe-N-PPC. RDE tests were performed to obtain the linear scan voltammetry (LSV) curves and further study the ORR performance of the samples. Fe-N-PPC displayed an onset potential of 0.966 V (Figure 5b), which was higher than those of PPC (0.89 V), N-PPC (0.899 V), Fe-PPC (0.940 V), and 20% Pt/C (0.952 V) catalysts. The prominent ORR activity of Fe-N-PPC was confirmed by its positive half-wave potential (0.891 V) and large current density (5.077 mA·cm^−2^). Pt/C showed a positive half-wave potential (0.865 V) and a large current density (4.92 mA·cm^−2^). Fe-N-PPC showed superior ORR activities as compared to other Fe_2_N-based catalysts reported in the literature. The large limited current density of Fe-N-PPC may result from its porous structure with anchored Fe_2_N particles and the broad diffusion pathways for solvated O_2_ [38]. The superior onset potential of Fe-PPC versus porous carbon and N-PPC was originated from the presence of Fe and Fe-based systems. The more positive potential of Fe-N-PPC was produced by the presence of Fe and N co-doping systems, which increased the number of ORR active sites of carbon-based electrocatalysts, as confirmed by the XRD and Raman data. Besides, assessment of preparation process of Fe-N-PPC is compared to recently reported Fe_2_N/C catalysts and pomelo peel-derived carbon materials for ORR. Table 4 reveals that Fe-N-PPC showed excellent electrochemical property and was prepared only with pomelo peels, ammonia and FeCl_3_·6H_2_O.

The K–L plots (Figure 5d) of Fe-N-PPC, obtained from the LSV experiments by varying the rotation rates and a scan rate of 10 mV/s (Figure 5c), exhibited a good linearity and allowed us to determine the *n* of this catalyst. Fe-N-PPC showed *n* values of 4.06, 4.06, and 4.07, suggesting that a four-electron (4e^−^) hybrid pathway prevailed from −0.6 to −0.8 V during the ORR process.

Durability tests were performed for the Fe-N-PPC and commercial Pt/C catalysts at −0.25 V vs. Ag/AgCl in an O_2_-saturated 0.1 M KOH aqueous solution at a rotation rate of 400 rpm (Figure 5e). After 20,000 s of reaction, the cathodic current decreased to 79.6% and 54.6% of the initial baseline value for Fe-N-PPC and Pt/C, respectively. Fe-N-PPC protected by a carbon shell on Fe_2_N nanoparticles, which can show the better stability [29]. The tolerance to methanol crossover effects was also tested for Fe-N-PPC and Pt/C (Figure 5f). After the addition of a 3 M methanol solution, commercial Pt/C instantaneously decreased its current as a result of the oxidation of methanol on the electrode surface. In contrast, Fe-N-PPC retained 97.2% of its initial baseline activity after methanol addition, revealing a good tolerance to methanol crossover.

## 3. Materials and Methods

### 3.1. Synthesis of Samples

Raw pomelo peel was air-dried and ground into a fine powder (80-mesh sieve). The powder was carbonized at 800 °C for 5 h under an Ar flow. The resulting porous carbon material was washed with deionized water and an HCl solution, and finally oven dried under air at 80 °C overnight to produce PPC. 1 g of PPC was subsequently dispersed under stirring in a saturated FeCl_3_ solution overnight to allow adsorption saturation. A small amount of FeCl_3_·6H_2_O was encapsulated in the pores of the carbon material. The FeCl_3_-saturated PPC material was collected by filtration and oven dried at 80 °C for 12 h and subsequently calcined by heating it from room temperature to 900 °C (5 °C·min^−1^) for 2 h under an NH_3_ atmosphere to finally produce Fe-N-PPC (i.e., Fe_2_N/N-PPC). For comparison, the porous carbon material was carbonized at similar conditions to obtain N-PPC. Fe-PPC was obtained by heating FeCl_3_-saturated porous carbon in an Ar atmosphere.

### 3.2. Characterization

A Hitachi SU8010 microscope (Hitachi, Tokyo, Japan) was used for the SEM observations. TEM, EDS and HRTEM images of the samples were obtained on JEM-2100 (JEOL, Tokyo, Japan) and Tecnai G2 F20 (FEI, Hillsboro, OR, USA) microscopes, respectively. XRD experiments were conducted on a Bruker D8 Advance X-ray diffractometer with Cu Kα radiation (λ = 1.5406 Å, Bruker, Karlsruhe, Germany). An AMICUS/ESCA 3400 device (Kratos, Manchester, UK) was used to collect the XPS data using Mg Kα (12 kV, 20 mA) radiation. The C1s line at 284.8 eV served as the reference. A Micromeritics ASAP 2020 N_2_ adsorption/desorption apparatus (Micromeritics, Atlanta, GA, USA) was used for performing the Barrett-Joyner-Halenda (BJH) pore structure analysis, and for measuring the BET specific surface area (SSA) at 77.35 K. Elemental mapping was conducted on a JEM-ARM 200F microscope operating at 200 kV (JEOL, Tokyo, Japan). The Raman spectra were obtained on a Renishaw instrument (Renishaw, London, UK) with an Ar ion laser (514.5 nm excitation wavelength).

### 3.3. Electrochemical Measurements

The ORR catalytic activity of the samples was evaluated on a CHI760D instrument (CH Instruments, Shanghai, China). The powder (5 mg) sample was mixed with a Nafion solution (5%) and ultrasonically mixed for 30 min before being loaded on a glassy carbon (GC) electrode (0.153 mg cm^−2^). The GC electrode containing a thin powder layer was fixed on a rotating disk electrode (RDE) that served as the working electrode (WE). Pt foil and Ag/AgCl served as counter and reference electrodes (CE and RE, respectively). A 0.1 M KOH aqueous solution served as the electrolyte, and the rotation speed varied from 400 to 2025 rpm. The potentials (versus Ag/AgCl) were converted to a RHE scale by the equation:

*E*_RHE_ = *E*_Ag/AgCl_ + 0.059 *pH* + 0.197 (1)

Koutecky-Levich (K–L) plots were subsequently obtained from the linear sweep voltammetry (LSV) data with the aim of measuring the electron transfer number (*n*) in the ORR process. The electron transfer number per O_2_ molecule in the ORR process can be calculated according to the following equations [39]:

*B* = 0.62*nFC*_o_ (*D*_o_)^2/3^ν^−1/6^ (2)

1/*J* = 1/*J_L_* + 1/*J_K_* = 1/*Bw*^1/2^ + 1/*J_K_* (3)

where *F* is the Faraday constant (96,485 C), *C*_o_ is the oxygen bulk concentration (1.2 × 10^−3^ mol·cm^−3^), *D*_o_ is the O_2_ diffusion coefficient (ca. 1.9 × 10^−5^ cm^2^·s^−1^), ν is the electrolyte kinematic viscosity (1.1 × 10^−2^ cm^2^·s^−1^), *J* is the measured current density, *ω = 2πN* with N being the linear rotation speed, *J_L_* is the diffusion-limiting current density, and *J_K_* is the kinetic current density.

## 4. Conclusions

In summary, N-doped highly graphitized porous carbon-supported Fe_2_N NPs (Fe-N-PPC) were successfully synthesized from pomelo peel, ammonia and FeCl_3_·6H_2_O via a two-step pyrolysis processes. The resulting Fe-N-PPC catalyst exhibited an outstanding ORR activity in alkaline media with onset and half-wave potentials of 0.966 and 0.891 V, respectively. These values were more positive than those of a commercial Pt/C catalyst. Fe-N-PPC also showed excellent electrochemical stability and methanol tolerance compared to Pt/C. These good characteristics resulted from the high specific surface area and the synergistic effect of Fe_2_N. In summary, the Fe-N-PPC catalyst represents a promising alternative to expensive commercial Pt/C catalysts for practical applications of alkaline fuel cells.

## Figures and Tables

**Table 1:** Atomic contents of the samples by X-ray photoelectron spectroscopy (XPS).

Samples	C	N	O	Fe
PPC	90.94	≈0	9.06	-
N-PPC	96.39	1.61	2.01	-
Fe-PPC	96.21	-	3.27	0.53
Fe-N-PPC	95.86	1.60	2.21	0.43

**Table 2:** Atomic ratio of N species from deconvolution N 1s spectra.

Samples	Pyridinic	Fe-N	Pyrrolic	Graphitic	Oxidized
PPC	25.9	-	15.0	34.5	24.6
N-PPC	31.9	-	10.8	32.2	25.1
Fe-N-PPC	32.2	9.0	12.8	33.6	12.4

**Table 3:** BET surface area and Barrett–Joyner–Halenda (BJH) pore diameter of PPC and Fe-N-PPC.

Sample	S_BET_ (m^2^·g^−1^)	D_BJH_ (nm)	Pore Volume (cm^3^·g^−1^)
PPC	353.05	2.60	0.23
Fe-N-PPC	1103.90	3.48	0.96

**Table 4:** Comparation of Fe-N-PC with Fe_2_N/C catalysts and pomelo peel-derived carbon materials for oxygen reduction reaction (ORR) reported recently. In the table, reversible hydrogen electrode was abbreviated as RHE. GO stands for graphene oxide.

Catalysts	Raw Materials	Onset Potential (V vs. RHE)	Half-Wave Potential (V vs. RHE)	Stability Result (%)	Methanol Tolerance Result (%)	Reference
ZIF-67@NPC-2	Pomelo peel, cobalt(II) acetate, methyl-imidazole	0.89	0.82	82.0 (6000 s)	95.7 (3 M)	[16]
Fe_3_C/WC/GC	Pomelo peel, K_4_Fe(CN)_6_, Na_2_WO_4_	-	-	-	-	[17]
Fe_2_N/MNGCS	Dopamine, NH_3_·H_2_O	0.881	-	95.2 (60,000 s)	70.7 (4 M)	[20]
FeGH-ArNH_3_	Fe_3_O_4_, histidine, Fe(NO_3_)_3_·9H_2_O, d-glucose	0.80	-	94.4 (6000s)	>Pt/C	[24]
Fe_x_N/NGA	GO, iron (II) phthalocyanine	>Pt/C	-	91.0 (20,000 s)	~100	[25]
Fe_2_N/N-GAs-20	FeCl_3_·6H_2_O, sodium alginate, graphene	1.02	0.93	90.0 (9 h)	~100	[29]
Fe_2_N@NPC-500	1,3,5-benzenetricarboxlic acid, *N*,*N*-Dimethylformamide, Fe(NO_3_)_3_·3H_2_O	0.93	0.79	90.0 (25,000 s)	~100	[33]
Fe–N-CNBs	FeCl_3_, fructose, magnesium powder, magnesium ribbon	1.03	0.875	83.0 (30,000 s)	~100	[34]
Fe_2_N/NGC-1000	FeCl_2_, polyacrylic weak-acid cation-exchanged resin	0.83	0.95	-	-	[37]
Fe-N-PPC	Pomelo peel, FeCl_3_·6H_2_O, ammonia	0.966	0.891	79.6 (20,000 s)	97.2 (3 M)	This work
